# Unusual Ventilatory Response to Exercise in Patient with Arnold-Chiari Type 1 Malformation after Posterior Fossa Decompression

**DOI:** 10.1155/2016/8359838

**Published:** 2016-06-21

**Authors:** Keely Smith, Ana M. Gomez-Rubio, Tomika S. Harris, Lauren E. Brooks, Ricardo A. Mosquera

**Affiliations:** ^1^Department of Pediatrics, McGovern Medical School, University of Texas Health Science Center at Houston, Houston, TX 77030, USA; ^2^McGovern Medical School, University of Texas Health Science Center at Houston, Houston, TX 77030, USA

## Abstract

We present a case of a 17-year-old Hispanic male with Arnold-Chiari Type 1 [AC-Type 1] with syringomyelia, status post decompression, who complains of exercise intolerance, headaches, and fatigue with exertion. The patient was found to have diurnal hypercapnia and nocturnal alveolar hypoventilation. Cardiopulmonary testing revealed blunting of the ventilatory response to the rise in carbon dioxide (CO_2_) resulting in failure of the parallel correlation between increased CO_2_ levels and ventilation; the expected vertical relationship between PETCO_2_ and minute ventilation during exercise was replaced with an almost horizontal relationship. No new pathology of the brainstem was discovered by MRI or neurological evaluation to explain this phenomenon. The patient was placed on continuous noninvasive open ventilation (NIOV) during the day and CPAP at night for a period of 6 months. His pCO_2_ level decreased to normal limits and his symptoms improved; specifically, he experienced less headaches and fatigue during exercise. In this report, we describe the abnormal response to exercise that patients with AC-Type 1 could potentially experience, even after decompression, characterized by the impairment of ventilator response to hypercapnia during exertion, reflecting a complete loss of chemical influence on breathing with no evidence of abnormality in the corticospinal pathway.

## 1. Introduction

Arnold-Chiari Type 1 (AC-Type 1) malformation is a complex syndrome in which the brainstem, medulla, and cerebellar tonsils herniate through the posterior fossa into the cervical spinal canal. Compression of the brainstem structures, including the respiratory center and its neural circuits, results in a wide variety of symptoms related to breathing such as sleep hypoventilation, central sleep apnea, and respiratory failure [1]. Medical literature suggests that most respiratory symptoms related to AC-Type 1 resolve after surgical decompression of the foramen magnum [1, 2]. However, in spite of surgical intervention, some patients continue to experience adverse respiratory symptoms [3].

During normal exercise, there is a physiologic increase in both oxygen consumption and carbon dioxide (CO_2_) production until the anaerobic threshold, which is typically about 60% of the subject's maximum work capacity, is reached. At this point, anaerobic metabolism leads to an increase in CO_2_ production compared to oxygen consumption. However, the arterial pCO_2_ level remains constant because the increase of CO_2_ stimulates central chemoreceptors in the medulla, triggering an increase in ventilation, thereby preventing hypercapnia [4, 5].

In this case report, we present a patient with AC-Type 1 with syringomyelia complaining of fatigue, headache, and somnolence during and after exercise. In addition, impairment of ventilator response to hypercapnia (baseline 60–70) during exertion was shown, reflecting a complete loss of chemical influence on breathing with no evidence of abnormality in the corticospinal pathway.

## 2. Case Presentation

A 17-year-old Hispanic male with a history of multiple medical and surgical problems associated with AC-Type 1 malformation and syringomyelia, status after surgical decompression, was referred to our Pulmonary Clinic for evaluation of respiratory symptoms. He presented with symptoms of exercise intolerance characterized by headaches, fatigue, and somnolence during and after exercise. In addition, he reported chronic symptoms of fatigue, irritability, increased daytime somnolence, early morning headaches, and disordered sleep breathing.

A thorough evaluation was performed. Arterial blood gas revealed the following results: pH of 7.31, pCO_2_ of 63 mmHg, pO_2_ of 93, HCO_3_ of 32 mMol/L, and a normal O_2_ saturation at 96%. Nocturnal polysomnography demonstrated end-tidal CO_2_ as high as 61 mmHg and CO_2_ for greater than 50 torr for 99% of total sleep time (tst), no apnea, and 13 hypopneas/hr. Spirometry at rest showed normal values: Forced-Volume Capacity (FVC) 117%, forced-expiratory volume 1 second (FEV1) 121%, FEV1/FVC 104%, and forced-expiratory flow (FEF) Max 115%.

The patient underwent a symptom-limited incremental cardiopulmonary exercise test (CPET). The patient was exercised on an electronically braked cycle ergometer using 20-watt incremental protocol while heart rate, blood pressure, tidal volume, minute ventilation, oxygen saturation, oxygen consumption, CO_2_ production, and perceptive response to exercise were monitored.

At baseline, the following values were recorded: respiratory rate (RR) was 10 BPM, tidal volume (*V*_*T*_) was 0.57 Lm, minute ventilation (Ve) was 5.9 L/min, SpO_2_ was 96%, VO_2_ was 3.8 mL/kg/min, heart rate (HR) was 97 bpm, blood pressure (BP) was 105/63, and PETCO_2_ was 45 mmHg (Table 1). After exercising for 9 minutes, the patient achieved anaerobic threshold, at which point the partial pressure of exhaled carbon dioxide (PETCO_2_) increased to 55 mmHg but Ve increased to only 31.5 L/min. The patient reached VO_2_ Max at 12 minutes after starting exercise, while working at a rate of 177 watts and a speed of 66 RPM, at which point the PETCO_2_ remained at 55 mmHg and Ve increased to 79.9 L/min (50% of predicted value).

The test also revealed normal values with regard to heart rate and rhythm, oxygen pulse response, and blood pressure. No abnormalities of heart rhythm or ST segment were noted and the patient did appear to have a low aerobic capacity and normal exercise efficiency. His maximum exercise was not limited by ventilation, and his breathing reserve was within normal values.

Evaluation by a neurologist revealed normal deep tendon reflexes, gait, and motor tone, ruling out suspicions of corticospinal tract abnormality. Additionally, his MRI was normal, showing that his brainstem structures were no longer compressed.

The patient was treated with 6 months of noninvasive open ventilation (NIOV) during the day and was placed on CPAP at night. NIOV was discontinued after 6 months of treatment, and his pCO_2_ was obtained. At this point, his venous pCO_2_ was within the normal limit (50 mmHg). Two months after NIOV was discontinued, the pCO_2_ remained normal (42 mmHg). In addition, the cardiopulmonary test was repeated and showed a slight improvement in ventilatory response. Specifically, the PETCO_2_ started at 41 mmHg and stayed at 41 mmHg at peak exercise (VO_2_ Max). An increase in Ve and Ve/VCO_2_ was also observed compared to before treatment.

## 3. Discussion

We present a teenage male with AC-Type 1 with exercise-induced hypercapnia after decompressive surgery, who was treated with continuous noninvasive open ventilation (NIOV) for a period of 6 months in order to reset the respiratory central chemoreceptors. Treatment with NIOV improved both the diurnal and exercise-induced hypercapnia.

Individuals with Arnold-Chiari Type 1 could potentially have blunted ventilatory response to hypercapnia and hypoxemia, which suggests abnormalities of central chemoreceptors. The cause of central hypoventilation in patients with Chiari malformation is thought to be due to dysgenesis of neural structures or damage to the brainstem and cerebellum during the herniation, causing impairment of the respiratory centers [6]. However, the symptoms of diurnal and exercise-induced hypercapnia, sleep disordered breathing, and vocal cord dysfunction persisted in our patient, despite successful surgical decompression and normal MRI.

The ventilatory response to constant work-rate exercise consists of 3 phases involving multiple mechanisms. The ventilatory response that occurs at the beginning of exercise is characterized by an immediate increase in ventilation. The next phase consists of a slow increase in ventilation, ultimately reaching a final steady phase if the exercise is not too severe. Ventilatory parameters in this phase show an increase in minute ventilation [Ve] due to both tidal volume [*V*_*T*_] and respiratory rate [RR]. Initially, the increase in *V*_*T*_ exceeds the respiratory rate. However, as metabolic acidosis develops, the respiratory rate predominates. The mechanism by which minute ventilation increases during this phase remains controversial. At mild to moderate exercise, characterized by exercise levels not reaching lactic acidosis, there is an increase in ventilation primarily driven by arterial chemoreceptors which respond to oscillatory changes in the blood gases, despite the PaCO_2_ and PaO_2_ levels [5].

**Figure 1:**
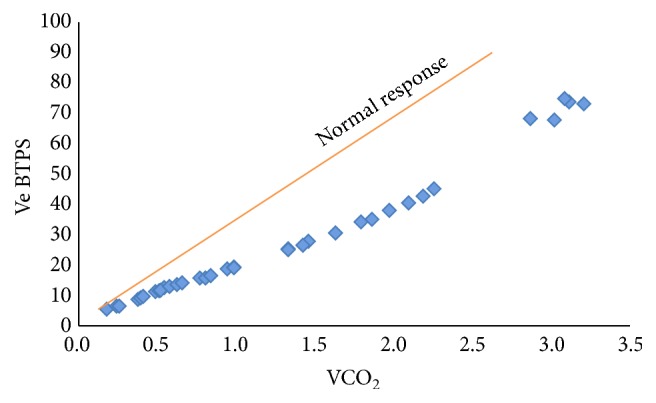
Relationship between Ve and VCO_2_ before treatment.

In our patient, the level of PETCO_2_ significantly increased during exercise. In addition, Ve did not increase as expected most likely due to an abnormality in the central chemoreceptors, which is associated with lower Ve (Figure 1). It is known that, in patients with progressive neuromuscular degenerative disease, respiratory compromise leads to disordered breathing and chronic hypercapnia, which may be alleviated by resetting the sensitivity of the central respiratory centers with noninvasive open ventilation for at least four hours a day [7]. With this concept in mind, the patient was placed on 6 months of NIOV which improved both his baseline pCO_2_ and his exercised induced hypercapnia. These effects persisted even 2 months after NIOV was discontinued. We speculate that the respiratory central chemoreceptors were reset.

This case underscores the importance of a thorough evaluation of the respiratory drive, including cardiopulmonary exercise testing, in patients with AC-Type 1 with chronic symptoms of exercise intolerance including headaches, fatigue, somnolence, and hypercapnia.

## Figures and Tables

**Table 1:** Cardiopulmonary exercise test results.

	Baseline	Anaerobic threshold	VO_2_ Max
Ventilation			
RR	10 BPM	15 BPM	31 BPM
*V*_*T*_	0.57 L	2.13 L	2.56 L
Ve	5.9 L/min	31.5 L/min	79.9 L/min
SpO_2_	96%	96%	96%

O_2_ consumption			
VO_2_ (mL/kg/min)	3.8	22.7	34.6 (78% predicted)
VCO_2_	210	1671	3403
RER	0.81	1.08	1.43

Cardiac			
HR	97 bpm	158 bpm	194 bpm

V/Q			
Ve/VCO_2_	28	19	23
Ve/VO_2_	23	20	34
PETCO_2_	45 mmHg	56 mmHg	50 mmHg
PETO_2_	93 mmHg	91 mmHg	109 mmHg
